# Scleredema Diabeticorum with Superimposed Cellulitis and Abscess Formation

**DOI:** 10.1155/2018/9513768

**Published:** 2018-04-16

**Authors:** Binav Shrestha, Eliza Sharma, Osama Mukhtar, Jaspreet Kaler, Shivani Thapa, Mazin Khalid

**Affiliations:** ^1^Interfaith Medical Center, Brooklyn, NY, USA; ^2^Maimonides Medical Center, Brooklyn, NY, USA

## Abstract

Scleredema diabeticorum is a rare cutaneous manifestation of diabetes mellitus. We present a case of an obese male with poorly controlled diabetes who came to the hospital with upper back pain and subsequently developed sepsis due to a small deep-seated abscess in his back that was drained and treated with antibiotics. He was also found to have extensive induration of the skin over his back and neck. Skin biopsy confirmed the diagnosis of scleredema diabeticorum.

## 1. Introduction

Diabetes mellitus (DM) is associated with different skin disorders. While skin infections and xerosis are the two most common manifestations, some form of cutaneous involvement is present in up to four-fifths of diabetic patients [1–3]. Some skin disorders like acanthosis nigricans are associated with insulin resistance while others like vitiligo are related to the autoimmune process in type 1 diabetes mellitus. These cutaneous manifestations can either be the initial presentation of diabetes or present at different time courses of the disease. Many skin lesions like bullosis diabeticorum and necrobiosis lipoidica have classically been considered specific for DM, though conflicting new shreds of evidence are seen [4–6]. Scleredema diabeticorum (SD) is a rare condition of unknown pathogenesis and is characterized by nonpitting thickening and induration of the skin [7, 8]. Here we present a case of SD in a patient with poorly controlled type 2 diabetes mellitus.

## 2. Case Report

**Figure 1:**
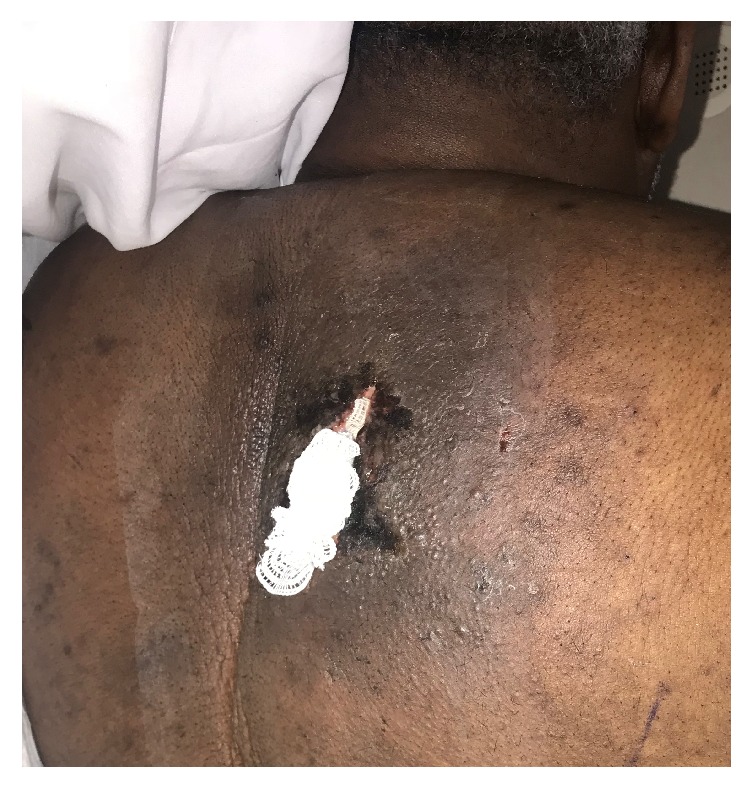
Upper back demonstrating thickened skin after incision and drainage.

**Figure 2:**
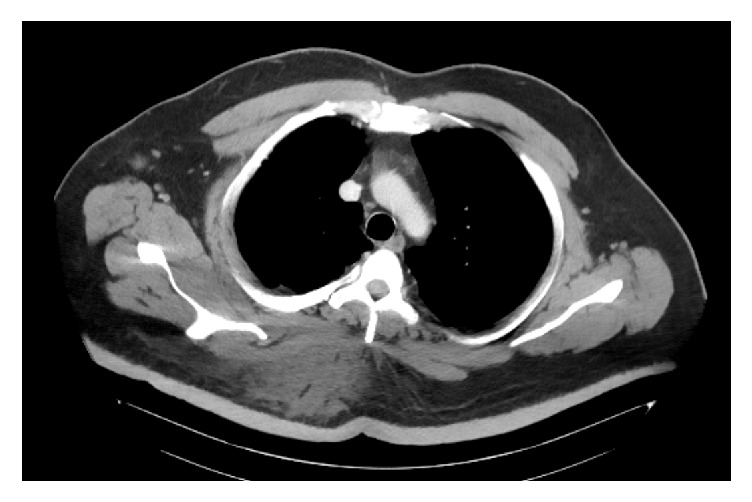
CT scan of the chest showing a large area of subcutaneous tissue density of right upper back.

**Figure 3:**
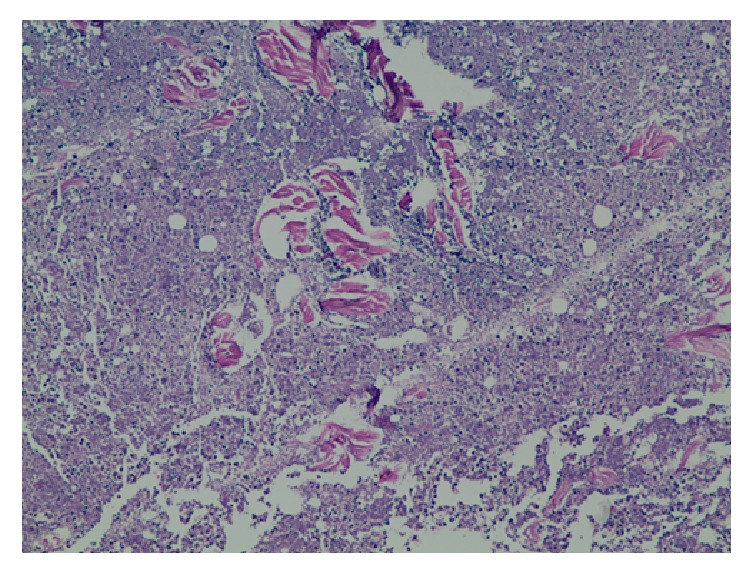
Histopathology from skin biopsy of the upper back showing fibroadipose tissue and skeletal muscle with focal acute and chronic inflammation.

A 54-year-old man presented with worsening sharp pain over his right upper back. The pain had started two weeks back immediately after getting a flu vaccination in his right shoulder. There was no radiation and no aggravating or relieving factors. It was associated with a localized sensation of warmth and occasional chills. He, however, denied any preceding trauma, fever, cough, weakness, numbness, or pain in any other part of the body. Rest of the review of symptoms was negative. His past medical history was significant for hypertension, type 2 DM, morbid obesity with a body mass index of 44 kg/m^2^, and depression. He was not compliant with his diet nor his medications. At presentation, he was afebrile but, subsequently, he developed a fever with a temperature of 102 F. His blood pressure was 137/75 mm Hg with a pulse rate of 113 beats per minute. His physical examination was significant for an extensive area of induration in the right upper back extending into the back of the neck, measured about 25 cm × 20 cm in size, and had a 3 cm × 4 cm × 3 cm area of fluctuation with erythema and tenderness (Figure 1). His laboratory investigations were significant for leukocytosis with a white blood cell count of 26,000/*μ*L, lactic acidosis with a serum lactic acid of 2.4 mmol/L (normal 0.5–1.9 mmol/L) and serum bicarbonate of 16 mmol/L (normal 22–32 mmol/L), and poorly controlled diabetes with serum glucose of 291 mg/dL (normal 74–118 mg/dL) and subsequent hemoglobin A1_C_ of 10.3% (normal 4.8%–5.6%). Erythrocyte sedimentation rate was 104 mm/hr (normal 0–20 mm/hr), C-reactive protein was 345.7 mg/L (normal 0–4.9 mg/L), thyroid stimulating hormone level was 1.360 *μ*IU/mL (normal 0.450–4.50 uIU/mL), and free thyroxine level was 5.1 *μ*g/dL (normal 4.5–12 ug/dL). Serum protein electrophoresis (SPEP) showed a normal pattern with no monoclonal protein, and antistreptolysin O (ASO) titer was negative. Chest imaging showed bullous changes in the upper lobes of the lung, a large area of soft tissue density in the subcutaneous tissue of right upper back, and enlarged right trapezius muscle (Figure 2). In this setting of fever, tachycardia, leukocytosis, and lactic acidosis, a presumptive diagnosis of sepsis likely due to cellulitis of the back with abscess formation was made. The patient was then started on broad-spectrum intravenous antibiotics and taken to the operative room for incision and drainage. His deep-seated abscess was drained and cultured, which subsequently grew* Staphylococcus lugdunensis* sensitive to methicillin, so the patient was started on intravenous Nafcillin. A skin biopsy taken from the back was sent for histopathology and showed fibroadipose tissue and skeletal muscle with focal acute and chronic inflammation with granulation tissue (Figure 3). These histopathological findings were consistent with scleredema diabeticorum and was thought to have resulted from his longstanding, poorly controlled diabetes. However, he denied prior symptoms of tightness or discomfort over the shoulders and upper back. The patient was counseled extensively and started on insulin and metformin, and the dosage was optimized during hospitalization. He was also informed about the treatment options for scleredema diabeticorum including immunosuppressants and phototherapies, but he declined any treatment for the time being. And with the sepsis resolved, blood glucose better controlled, and his surgical wound healing, he was discharged home to follow up as an outpatient.

## 3. Discussion

Scleredema is a sclerotic skin disease and is characterized by woody induration of the skin. Histologically, it is characterized by marked thickening of the dermis layer of the skin with thickened collagen bundles separated by mucin deposition. It mainly involves the skin of the upper part of the body, predominantly the back, neck, and shoulders. Three variants of scleredema have been described based on their association with streptococcal infection, monoclonal gammopathy, or diabetes mellitus [9, 10]. Our patient, however, had negative ASO titer with normal SPEP, and the findings were consistent with scleredema diabeticorum (SD).

SD is a rare manifestation of DM. It may, however, have been grossly underrecognized and the actual prevalence of SD in DM has been reported to be between 2.5% and 14% in a few studies [11, 12]. SD is mostly seen, like in our case, in obese patients with poorly controlled diabetes. The exact pathogenesis of SD remains unclear but is thought to result from irreversible nonenzymatic glycosylation of collagen fibers that impairs its degradation [13]. High levels of glucose are also hypothesized to stimulate fibroblast proliferation [14]. Most of the patients remain asymptomatic and unrecognized. It may also present with pain, erythema, or pigmentation, while severe cases may cause a restrictive pulmonary defect or impaired mobility of joints [15]. And like in our patient, it can be superimposed with superficial as well as deep-seated cutaneous and subcutaneous infections. Various treatment modalities, including immunosuppressants like corticosteroids and methotrexate, electron beam radiotherapy, tamoxifen, psoralen plus ultraviolet A (PUVA) therapy, and ultraviolet A1 phototherapy, have been used for SD with mixed and inconsistent results [16–21]. Therefore, in most cases of scleredema diabeticorum like our patient, tighter glycemic control remains the first and foremost means of therapy [22].

## 4. Conclusion

Scleredema diabeticorum is a rare and frequently unrecognized manifestation of diabetes. It is mostly seen in patients with poor glycemic control. Physicians need to be aware of this entity for early recognition and prevention of complications like a restrictive pulmonary defect.

## References

[1] Demirseren D. D., Emre S., Akoglu G. (2014). Relationship between skin diseases and extracutaneous complications of diabetes mellitus: Clinical analysis of 750 patients. *American Journal of Clinical Dermatology*.

[2] Goyal A., Raina S., Kaushal S., Mahajan V., Sharma N. (2010). Pattern of cutaneous manifestations in diabetes mellitus. *Indian Journal of Dermatology*.

[3] Romano C., Massai L., Asta F., Signorini A. M. (2001). Prevalence of dermatophytic skin and nail infections in diabetic patients. *Mycoses*.

[4] Muller S. A., Winkelmann R. K. (1966). Necrobiosis Lipoidica Diabeticorum: A Clinical and Pathological Investigation of 171 Cases. *JAMA Dermatology*.

[5] Cantwell A. R., Martz W. (1967). Idiopathic bullae in diabetics. Bullosis diabeticorum. *JAMA Dermatology*.

[6] O'Toole E. A., Kennedy U., Nolan J. J., Young M. M., Rogers S., Barnes L. (1999). Necrobiosis lipoidica: Only a minority of patients have diabetes mellitus. *British Journal of Dermatology*.

[7] Krakowski A., Covo J., Berlin C. (1973). Diabetic scleredema. *Dermatology*.

[8] Toyota T., Umezu M., Oikawa N. (1983). Diabetic Scleredema. *The Tohoku Journal of Experimental Medicine*.

[9] Graff, R., *Discussion.* Arch Dermatol, 1968. 98: p. 320

[10] Kovary P. M., Vakilzadeh F., Macher E., Zaun H., Merk H., Goerz G. (1981). Monoclonal gammopathy in scleredema. Observations in three cases. *JAMA Dermatology*.

[11] Cole G. W., Headley J., Skowsky R. (1983). Scleredema diabeticorum: A common and distinct cutaneous manifestation of diabetes mellitus. *Diabetes Care*.

[12] Sattar M. A., Diab S., Sugathan T. N., Sivanandasingham P., Fenech F. F. (1988). Scleroedema Diabeticorum: a Minor but Often Unrecognized Complication of Diabetes Mellitus. *Diabetic Medicine*.

[13] Twigg S. M., Chen M. M., Joly A. H. (2001). Advanced glycosylation end products up-regulate connective tissue growth factor (insulin-like growth factor-binding protein-related protein 2) in human fibroblasts: A potential mechanism for expansion of extracellular matrix in diabetes mellitus. *Endocrinology*.

[14] Haustein U.-F. (1999). Scleroderma-like lesions in insulin-dependent diabetes mellitus. *Journal of the European Academy of Dermatology and Venereology*.

[15] Venencie P. Y., Powell F. C., Su W. P. D., Perry H. O. (1984). Scleredema: A review of thirty-three cases. *Journal of the American Academy of Dermatology*.

[16] Thumpimukvatana N., Wongpraparut C., Lim H. W. (2010). Scleredema diabeticorum successfully treated with ultraviolet A1 phototherapy. *The Journal of Dermatology*.

[17] Hager C. M., Sobhi H. A., Hunzelmann N. (1998). Bath-PUVA therapy in three patients with scleredema adultorum. *Journal of the American Academy of Dermatology*.

[18] Alsaeedi S. H., Lee P. (2010). Treatment of scleredema diabeticorum with tamoxifen. *The Journal of Rheumatology*.

[19] Janiga J. J., Ward D. H., Lim H. W. (2004). UVA-1 as a treatment for scleredema. *Photodermatology, Photoimmunology & Photomedicine*.

[20] Dogra S., Handa S., Kanwar A. J. (2004). Dexamethasone pulse therapy for scleredema [5]. *Pediatric Dermatology*.

[21] Bowen A. R., Smith L., Zone J. J. (2003). Scleredema adultorum of Buschke treated with radiation. *JAMA Dermatology*.

[22] Baillot-Rudoni S., Apostol D., Vaillant G., Brun J.-M., Renard E. (2006). Implantable pump therapy restores metabolic control and quality of life in type 1 diabetic patients with Buschke's nonsystemic scleroderma [1]. *Diabetes Care*.

